# Knock, knock, who is there? Two studies provide new insights into the translocation of pathogen effectors into plant cells

**DOI:** 10.1093/plcell/koad097

**Published:** 2023-03-28

**Authors:** Mariana Schuster

**Affiliations:** Assistant Features Editor, The Plant Cell, American Society of Plant Biologists, USA; Leibniz Institute of Plant Biochemistry, 06120, Halle (Saale), Germany

Plant pathogens secrete versatile virulence proteins called effectors to manipulate plant processes that are crucial for infection. Pathogen effectors can act either outside of plant cells (apoplastic effectors) or within the host cell (cytoplasmic effectors). Bacterial phytopathogens are known to use specialized, multiprotein secretion systems to translocate their effectors into host cells (Chang et al. 2014). For filamentous plant pathogens, including fungi and oomycetes, evidence suggesting the involvement of a multiprotein complex for effector translocation into plant cells, has only recently been shown for the maize pathogenic fungus *Ustilago maydis* (Ludwig et al. 2021). In other filamentous plant pathogens, invasive hyphae and membrane-rich structures associated with infection, such as haustoria or the biotrophic interfacial complex (BIC), have been implicated in the secretion of cytoplasmic effectors (Giraldo et al. 2013; Wang et al. 2017). But, the mechanism of effector translocation by this important class of plant pathogens, remains unknown.

In this issue of *The Plant Cell,* two back-to-back studies shed light on the mechanism by which filamentous plant pathogens exploit host clathrin-mediated endocytosis (CME) to translocate effectors into plant cells. In the first study, **Haixia Wang and coauthors** (Wang et al. 2023) focus on *Phytophthora infestans*, the oomycete pathogen causing potato late blight disease. Wang et al. investigated the translocation of a prominent class of cytoplasmic effectors, known as “RXLR” effectors, which are defined by a conserved *N*-terminal RXLR (Arg-Xaa-Leu-Arg) motif. In the second study, **Ely Oliveira-Garcia and co-authors** (Oliveira-Garcia et al. 2023) investigated the blast fungus *Magnaporthe oryzae*, which infects a wide range of plants, including rice. These researchers focused on the translocation of cytoplasmic effectors secreted at the BIC.

CME is the primary mechanism by which extracellular molecules enter plant cells. During CME, a complex comprising clathrin heavy chain (CHC), clathrin light chain (CLC), and adaptor proteins assemble at the plasma membrane. This complex, along with additional proteins, forms a grid that covers and pinches off a part of the plasma membrane, forming clathrin-coated vesicles (CCVs) that contain both a portion of the plasma membrane and the extracellular cargo. Subsequently, CCVs fuse with the trans-Golgi network/early endosomes (TGN/EEs), a process regulated by Rab GTPases, to release their cargo (Chen et al. 2011). The TGN/EEs act as a sorting station, where the cargo is sorted for further processing, recycling, or degradation.

To investigate the involvement of CME in cytoplasmic effector translocation, both Oliveira-Garcia et al. and Wang et al. used confocal microscopy to access the association of cytosolic effectors with key components of CME in the respective host plants rice and *Nicotiana benthamiana*. Additionally, they silenced CME components to evaluate their role in cytoplasmic effector translocation. In the *P. infestans* system, green fluorescent protein (GFP)-tagged versions of the clathrin light chain (NbCLC) and the plant-specific Rab GTPase Ara6 (NbAra6) were found to be associated with vesicles surrounding the haustorium. Silencing the genes encoding clathrin heavy chain (*NbCHC*) or *NbAra6* in planta reduced *P. infestans* colonization of *N. benthamiana* leaves (see Fig. A). Similarly, Oliveira-Garcia et al. generated rice transgenic lines expressing eGFP-tagged rice clathrin light chain (OsCLC) and discovered that the GFP signal colocalized with effector-labeled membrane structures that associate with the BIC. These individual membranous effector compartments (MECs) were shown to contain three different fluorescent-tagged cytoplasmic effectors from *M. oryzae*. Silencing genes encoding the CME components [adaptor protein complex-2 subunit 2α (Os*AP-2α*) and clathrin heavy chain-1 (Os*CHC1*)] also resulted in reduced pathogenicity of *M. oryzae* on rice (see Fig. B). These experiments suggest that in both pathosystems, pathogens exploit CME for cytoplasmic effector translocation.

**Figure.**
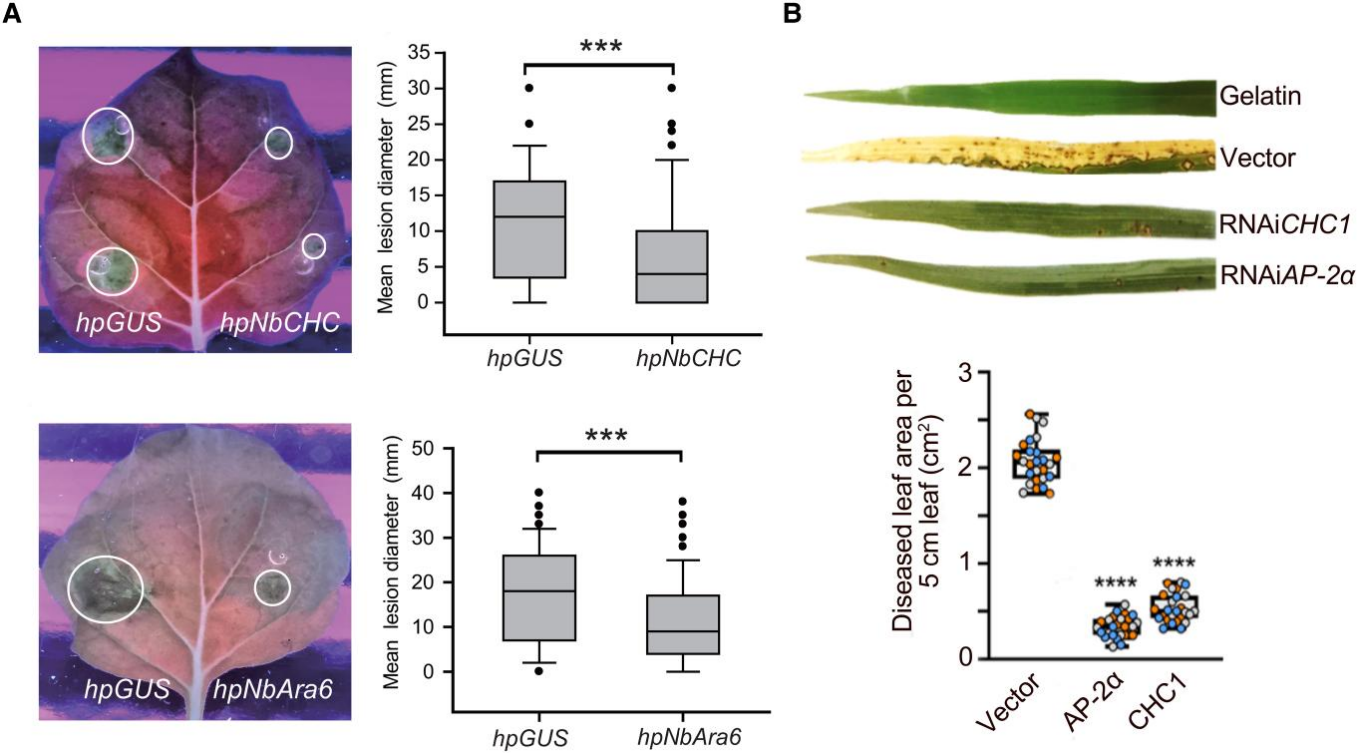
Silencing of clathrin mediated endocytosis in host plants reduces filamentous pathogen colonization. **A**) Silencing of *NbCHC* or *NbAra6* reduces *P. infestans* leaf colonization of *N. benthamiana* leaves. In transient coexpression experiments, infection with wild-type *P. infestans* 88069 is compromised in *NbCHC*-silenced (upper left) or *NbAra6*-silenced (lower left) leaf sectors compared to control sectors expressing a GUS-silencing construct (hpGUS). Leaf images taken at 8 dpi under UV light. Corresponding leaf lesion diameters are shown on the right. Box and whisker plots show 95% confidence intervals, and asterisks indicate a significant difference at *P* = 10-5 from paired 2-tailed t-tests. Adapted from Wang et al. (2023), Figure 1. **B**) Silencing of *AP-2α* or *CHC1* reduces *M. oryzae* colonization of rice leaves. In whole plant spray inoculation experiments, infection with wild-type *M. oryzae* strain Guy11 resulted in near disease abolishment in *CHC1*-silenced or *AP-2α-*silenced plants compared to the empty vector or gelatin controls. Corresponding quantification of diseased leaf area in 5 cm leaf segments is shown below. Box and whisker plots with individual data points are shown; data points in different colors represent different biological replicates (*P* < 0.0001 for all treatments; *n* = 9 rice plants per replication). Adapted from Oliveira-Garcia et al. (2023) Figure 7.

To directly investigate the involvement of clathrin and NbAra6 in RXLR effector translocation, Wang et al. infected *N. benthamiana* with a transgenic *P. infestans* strain carrying a red fluorescent protein (RFP)-tagged RXLR effector, Pi04314. Quantification of effector accumulation in plant cells revealed reduced RFP fluorescence in the nuclei of *NbCHC-* and *NbAra6-*silenced plants compared to the control. Similarly, in the rice pathosystem, silencing of either CME component *AP-2α* or *CHC1* using RNAi led to the absence of MECs. This observation is interpreted as effectors being trapped outside the invasive hyphae and unable to translocate. This phenotype was also observed by blocking CME with chemical inhibitors. Finally, Wang et al. used complementary biochemical methods to validate and expand on the CME-mediated effector translocation mechanism. They isolated and studied clathrin and NbAra6-associated endosomes, demonstrating that when isolated from infected material, they predominantly contained RXLR effectors, but not apoplastic effectors or other abundant *P. infestans* proteins.

These two reports shed new light on the translocation of cytoplasmic effectors into plant cells by hijacking the conserved CME pathway of the host. These findings provide valuable insights into the long-sought-after mechanism of effector translocation, which could be harnessed for the development of biotechnological solutions to mitigate or prevent the devastating diseases caused by filamentous pathogens.
